# An Advanced IoT-based System for Intelligent Energy Management in Buildings

**DOI:** 10.3390/s18020610

**Published:** 2018-02-16

**Authors:** Vangelis Marinakis, Haris Doukas

**Affiliations:** Decision Support Systems Laboratory, School of Electrical & Computer Engineering, National Technical University of Athens, 9, Iroon Polytechniou str., 157 80 Athens, Greece; h_doukas@epu.ntua.gr

**Keywords:** IoT, semantic web, rules, energy efficient, smart building, smart city

## Abstract

The energy sector is closely interconnected with the building sector and integrated Information and Communication Technologies (ICT) solutions for effective energy management supporting decision-making at building, district and city level are key fundamental elements for making a city Smart. The available systems are designed and intended exclusively for a predefined number of cases and systems without allowing for expansion and interoperability with other applications that is partially due to the lack of semantics. This paper presents an advanced Internet of Things (IoT) based system for intelligent energy management in buildings. A semantic framework is introduced aiming at the unified and standardised modelling of the entities that constitute the building environment. Suitable rules are formed, aiming at the intelligent energy management and the general modus operandi of Smart Building. In this context, an IoT-based system was implemented, which enhances the interactivity of the buildings’ energy management systems. The results from its pilot application are presented and discussed. The proposed system extends existing approaches and integrates cross-domain data, such as the building’s data (e.g., energy management systems), energy production, energy prices, weather data and end-users’ behaviour, in order to produce daily and weekly action plans for the energy end-users with actionable personalised information.

## 1. Introduction

One of the major challenges that the European Union (EU) faces within the scope of sustainable development is the increasing energy demand patterns of cities [1]. European cities should be places of advanced social progress and environmental regeneration, as well as places of attraction and engines of economic growth, based on a holistic integrated approach in which all aspects of sustainability are taken into account [2].

Cities are faced with a number of challenges associated with accommodation, atmosphere, transport and infrastructural development, making difficult for urban communities and cities to realise their objectives. In recent years, cities have been turning to advanced technologies to become Smart Cities. This term is used to describe Information and Communication Technological (ICT) solutions for cities and to highlight ICT importance and potential in helping the city to develop competitive advantages. More specifically, Smart Cities are comprised of cities that work in frugal and sound ways, by incorporating every one of its substructure and administrations into a unified whole and utilising insightful gadgets for observing and control, in order to guarantee maintainability and effectiveness [3,4].

Energy demand is one of the most crucial and multifaceted problems for Smart Cities [5,6]. As the quality of life is being improved, as well as the continuous increase of the population is given, it is obvious that the increase in energy demand is an irreversible situation. This continuous increase in energy demand coupled with limited conventional energy reserves are the main factors contributing to the increase in energy problems, which every city will have to resolve. Meanwhile, the ever increasing energy demand, combined with the human tendency towards constant enhancement of the standard of living, have resulted in the automation of a multitude of laborious and tedious tasks [7].

A wide set of measures has been adopted across individual Member States (MS) to actively optimise energy use, especially in buildings that account for 40% of total energy consumption in the EU [8]. The Energy Performance of Buildings Directive (EPBD) is the main policy driver at the EU level to support energy savings at the building sector. EPBD was adopted at the EU level in 2002 (2002/91/EC) and was recast in 2010 (31/2010/EU) with more ambitious provisions (EC, 2002; 2010). The EPBD introduction has already enabled many EU countries to be more active in the area of buildings’ energy performance and many others that had already defined requirements and relative frameworks to better understand and improve the status of their building stock. On 30 November 2016, the Commission proposed an update to the EPBD to help promote the use of smart technology in buildings and to streamline the existing rules. The Commission also published a new buildings database—the EU Building Stock Observatory—to track the energy performance of buildings across Europe [9].

A corollary of all the above was the emergence of the vision of the Smart Building as an environment that combines ambient intelligence and home automation, in order to enable the provision of high-level services to the residents that will ensure increasing comfort and safety within the house, as well as energy efficiency and rational management of resources [10,11]. At the same time, reliability and flexibility offered by wireless technologies have been the driving force for turning the Smart Building market towards the vision of the Internet of Things (IoT) and have contributed to attracting growing interest in the market. The introduction of IoT in energy and the methods using “intelligent” energy management and Internet technologies constitute an important factor in promoting efficient energy and environmental management of the “smart” building [12]. In particular, the connection of internet technologies in the energy has already created a new emerging market for energy services.

It is of common understanding, however, that achieving energy savings in buildings is a difficult and complex process [13,14]. Integrated, transparent and comprehensive approaches are required to provide cities the tools and methods they need to achieve significant reduction of energy consumption and CO_2_ emissions through the contribution of advanced ICT tools [15,16]. Indeed, although there is plenty of energy related data available in the cities, the need for methodologies and validated tools to collect, integrate and analyse them, supporting energy use management, is highlighted [17,18].

Nevertheless, the proposed systems are designed and intended exclusively for a predefined number of cases and systems without allowing for expansion and interoperability with other applications, which is partially due to the lack of semantics. Semantic Web advances give a way to share information about urban areas as physical, social, and specialised frameworks, thus empowering the development of shrewd city employments. The attributes of Smart Cities indicate that they apply the technological data to make effective the usage of infrastructural development that are physical in nature including the environment that is built, roads, and assets [19]. Semantic technologies have been used to create models of urban energy systems able to assess the energy performance of an urban area [20]. Lastly, they enable learning, adaptation and innovation by responding more efficiently and quickly to the varying situations through improvement of the city’s intelligence.

The Smart Building market is undoubtedly undergoing a fundamental shift towards the exploitation of wireless technologies and is focusing primarily on implementing the vision of IoT. The differentiation and heterogeneity of the offered solutions in levels of both hardware and software diverge from the basic principles of the IoT that require the use of a standard unified model, in order for maximum functionality to be ensured.

This paper presents an advanced IoT-based system for intelligent energy management in buildings. More specifically, a semantic framework for the unified and standardized modelling of all entities that constitute the environment of Smart Buildings, as well as their properties and relations, is proposed. This semantic modelling aims to be a realistic and alternative approach that is expected to resolve many of the current issues faced by the Smart Buildings market, as well as to improve knowledge reasoning and decision-making. Suitable rules are formed, aiming at the intelligent energy management and the general modus operandi of Smart Building. In this context, a web-based tool was implemented, which enhances the interactivity of buildings’ energy management systems. The proposed tool collects, stores and represents in real-time the energy data of buildings. Based on real-time data (from heterogeneous and dynamic sources: building’s data, energy production, energy prices, weather data and end-users’ behaviour), as well as predicted data produced by prediction models (renewable energy production, energy consumption, indoor temperature and energy prices), the tool introduces a list of practical action plans for the buildings’ occupants, structured upon a number of rules. The results from its pilot application are presented and discussed.

Apart from the introduction, the paper is structured along five sections. A review of the current state of the art, as well as the actual contribution of the proposed IoT-based system, is provided in Section 2. The internal architecture and the key features of the system (five data capturing modules, semantic framework and action engine) are presented in Section 3. Section 4 is devoted to the presentation of the proposed IoT-based system. Section 5 is devoted to the pilot application. Finally, the last section is summarizing the key issues that have arisen in this paper.

## 2. Tools and Methods

### 2.1. Literature Review

A number of available tools can support energy end-users in monitoring, managing and optimising their energy consumption. An innovative energy-aware IT ecosystem was presented by Fotopoulos et al. [21], providing personalized energy management and awareness services towards occupants’ behavioural change. Moreover, an IoT Energy Platform has been developed for the management of IoT energy data [22]. Related products from leading companies in the market are the following:

- Schneider Electric StruxureWare™ [23] is a platform of open, interoperable, and scalable software applications that provides energy managers with enterprise, operations or control level responsibility to optimise energy usage.
- Honeywell Attune Advisory Services enable on-going monitoring and optimisation of building energy performance. Attune is powered by cloud-based and Software as a Service (SaaS) technologies and energy and automation experts, which help facilities to determine how to best save energy, time and money [24].
- Siemens Synco™ is a control system for small and medium-size multipurpose buildings, such as shops, offices and apartments. The system primarily manages energy plants, controls and monitors the Heating, Ventilation, and Air Conditioning (HVAC) equipment in order to support the entire lifecycle of a building [25].
- Cylon Energy solution can be adapted to suit any type of building regardless of the Building Energy Management System or metering solution installed. It is based on a building energy monitoring system able to provide real-time (every 15 min) information on the energy usage and consumption in a building [26].

Other companies, such as eSight (Cambridge, UK), ENERIT (Galway, Ireland), DEXMA (Barcelona, Spain), as well as companies outside Europe, like BuildingIQ (San Mateo, CA, USA) and Ameresco (Framingham, MA, USA) have developed sector-specific tools for analytics and energy optimised solutions:

- eSight is an enterprise energy management software platform, 100% web-based, which offers different techniques for analysing energy usage and targeting areas to reduce energy consumption, costs and carbon by up to 30% [27].
- Enerit Systematic Energy Management Software promotes best practice energy management, offers complete coverage of IS0 50001 and aligns with Statement of Energy Performance (SEP) and Energy STAR [28].
- DEXCell Energy Manager is cloud-based and hardware-neutral (Manufacturer, City, US State abbrev. if applicable, Country). It combines advanced monitoring, analysis, alerts and reporting in an easy-to-use, scalable SaaS solution [29].
- Predictive Energy Optimization™ is Building IQ’s software platform, designed to improve the energy efficiency of large, complex commercial, public, and academic buildings [30].
- Ameresco’s Intelligent Solutions (AIS) energy data platform is comprised of a suite of services with its core energy efficiency offerings [31].

These tools are mostly used by energy/facility managers, Energy Service Company (ESCOs) and specialists, who make decisions based on the information they get. A number of start-ups and technology companies, like Loop Energy Saver (Woodbridge, UK) [32] and Origami Energy (London, UK) [33], NUUKA (Helsinki, Finland) [34] and OPTIWATTI (Espoo, Finland) [35], Plugwise [36] (Sassenheim, the Netherlands), SMARKIA (León, Spain) [37], as well as Bidgely (Sunnyvale, CA, USA) [38], Enetics (Canning Parkway Victor, NY, USA) [39] and PlotWatt (Durham, NC, USA) [40] provide tools that allow energy end-users in monitoring and managing their consumptions, or energy companies to reimagine customer engagement.

Some of the above-mentioned tools are energy management systems at the building level and others are modelling tools with functions that help optimising the energy systems. The available solutions focus mainly on energy data visualizations and notifications. In fact, these tools can apply and process just some of the input data elaborated by the proposed IoT-based System, to provide, in some cases, only monitoring and controlling activities, in others, energy analyses to help users make decisions on reducing energy consumption at the building level.

### 2.2. Adopted Approach

In Table 1, the entire operating process is represented, underlying the inputs received by the models and the benefits for the overall environment and for the single users that the action plans could bring.

The proposed system extends existing approaches and integrates cross-domain data, such as building’s data (e.g., energy management systems and other de-centralized sensor-based data), energy production, energy prices, weather data and end-users’ behaviour, in order to produce daily and weekly action plans for the energy end-users with actionable personalised information. These action plans are based on the data captured and short-term predictions of the user’s behaviour and energy usage. They include notifications for certain thresholds, analytical tailor-made recommendations and saving tips in the users’ daily routines (e.g., load shifting, occupancy, set-point adjustment).

The added value of the IoT-based System consists in correlating various types of real-time data from different sources, hence integrating different systems, in order to achieve intelligent energy management of buildings, and, potentially, districts. Moreover, the degree of generalization of the system makes this advanced tool easily adaptable to buildings/cities with different features regarding, for example, types of buildings, energy infrastructures and energy demand and not just focused on specific sectors or building targets. It brings together traditional monitoring systems, low-scale energy management systems and IoT practices, in order to achieve smart energy management.

Table 2 recaps the abovementioned systems, pointing out the types of input data applied.

## 3. Internal Architecture

**Figure 1:**
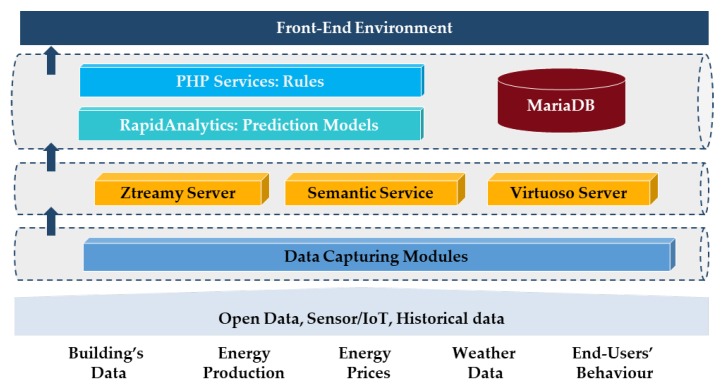
Internal architecture.

The proposed IoT-based System combines a series of components, as follows (Figure 1):

- Five data capturing modules, which collect data from different source (building’s data, energy production, energy prices, weather data and end-users’ behaviour).
- The semantic framework, which is a communication system that integrates data from multiple sources and domains using Semantic Web technologies.
- The action engine is an integrated solution for predicting the energy behaviour of buildings and to suggest actions to improve their energy efficiency. It can be integrated with existing middleware solutions to enhance them.

### 3.1. Data Capturing Modules

Specific data capturing modules has been developed for acquisition of site-specific data. Java or Python applications have been used for the development of the data capturing modules.

- Decentralized sensors indicate the real-time conditions on the spot by providing measurements of specific parameters such as the energy consumption, indoor temperature and humidity, etc.
- The module for Renewable Energy Sources (RES) production informs on the current level of self-production of energy of the connected renewable energy systems.
- The weather forecast module is able to provide a comparison of the forecast and the actual field conditions, for the creation of real-time energy balances.
- The energy prices module gives indication on the actual costs applicable for those who can adjust their energy contract to the current tariffs.
- The occupants’ feedback module is intended to gather the feedback about the comfort conditions of the occupants and other energy-related issues.

### 3.2. Semantic Framework

The second part of the procedure involved a communication system that integrates data from multiple sources (monitoring systems, Web Services, CSV files, etc.) and domains, with the purpose of contextualizing them in specific contexts, using Semantic Web technologies. It is based on the publish-and-subscribe communication pattern. More specifically, it has been implemented with the Ztreamy system, a semantic service implemented as a Python application. This service processes and contextualizes the data acquired from multiple sources. The Semantic Framework uses the Virtuoso triple-store as a data repository.

**Figure 2:**
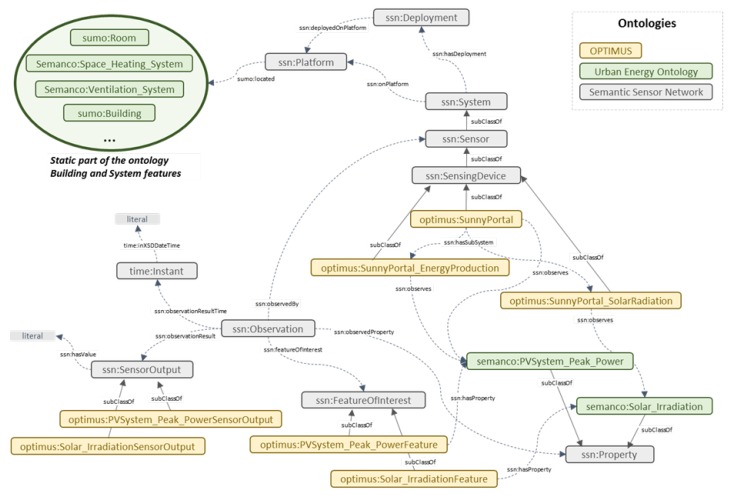
Ontology graph.

In this context, a relevant ontology was created (entitled OPTIMUS ontology) for all entities that are either included or related to the Smart Building environment and constitute the main vocabulary upon which the rules were based [41]. Figure 2 shows an excerpt of the OPTIMUS ontology referring to dynamic data, in particular to energy production sensors.

The ontology stands for a model of the static (e.g., building and technical systems features) and the dynamic (e.g., metering) characteristics of a building and their context (e.g., climate conditions and energy costs). In the field of ontology engineering, it is considered to be a good practice to reuse existing ontologies or vocabularies to avoid reinventing the wheel and to increase the interoperability of the ontology. The developed ontology is based on already existing ontologies, such as Urban Energy ontology [42] and Semantic Sensor Network ontology [43].

With respect to the static data, the Urban Energy ontology has been extended to model the building and technical system features, such as building geometry, building thermal envelope, Domestic Hot Water (DHW) systems, space cooling/heating systems, and energy generator. The concepts and properties that are not included in this ontology have been created in a new ontology called OPTIMUS.

The Urban Energy ontology has been chosen because it conceptualizes the same domain as the OPTIMUS ontology, and because it is based on existing energy information standards, including: ISO/IEC CD 13273 Energy efficiency regulation and renewable energy sources; ISO/DTR 16344 Common terms, the definitions and symbols for the overall energy performance rating and certification of buildings; ISO/CD 16346 Assessment of overall energy performance of buildings; ISO/DIS 12655 Presentation of real energy use of buildings; ISO/CD 16343 Methods for expressing energy performance and for energy certification of buildings; and ISO 50001:2011 Energy management systems–requirements with guidance for use [44].

Concerning the dynamic data, the Semantic Sensor Network (SSN) ontology has been extended to include different metering systems. The SSN ontology can describe sensors and observations. It is based on the Stimulus–Sensor–Observation ontology design pattern. In particular, this ontology includes capabilities, measurement processes, observations and deployments in which sensors are used [45]. The ontology is aligned with an upper ontology (i.e., Dolce Ultra Light ontology) and it is compatible with SensorML and O&M (Observations and Measurements) standards of the Open Geospatial Consortium. The SSN ontology describes sensors (i.e., ssn:Sensor) as physical objects that observe and transform incoming stimuli into another representation, where stimuli (i.e., ssn:Stimulus) are changes or states in an environment that a sensor uses to measure a property and where observations (i.e., ssn:Observation) are contexts for interpreting incoming stimuli and fixing parameters such as time and location. Since the SSN ontology provides only core concepts, it needs to be extended with domain specific terms. The domain terms that already exist in the Urban Energy model have been used while those that are not included in it have been created as concepts of the OPTIMUS ontology.

The Semantic Framework can be found on the central open source platform, Github [46]. The main contextual data added are the type of sensor (e.g. building’s data, energy production, energy prices, weather data and end-users’ behaviour) and properties observed (e.g. PVSystem_Peak_Power and Solar_Irradiation). The contextual triples are generated according to the OPTIMUS ontology. For each stream, the following parameters have to be configured:

- **stream**: Name of the stream.
- **owl_sensingdevice_class**: Class name of the sensor.
- **owl_sensingdevice_uri**: URI for identifying the sensor triples.
- **owl_observation_uri**: URI for identifying the observation triples.
- **owl_featureofinterest_uri**: URI for identifying the feature of interest triples.
- **owl_featureofinterest_class**: Class name of the Feature of Interest. It is usually the name of the observed property with ‘Feature’ string concatenated at the end.
- **owl_observedproperty_uri**: URI for identifying the property observed triples.
- **owl_observedproperty_class**: Class name of the property observed.
- **owl_sensoroutput_class**: Class name of the Sensor Output. It is usually the name of the observed property with ‘SensorOutput’ string concatenated at the end.

### 3.3. Action Engine

The action engine integrates prediction models, rules and a MariaDB database to store the results.

The prediction models are data-driven models to forecast the energy behaviour of a building according to some specific indicators (e.g., renewable energy production, energy consumption, indoor temperature and energy prices). The prediction models are automatically estimated and customized per building given the measure to be forecasted and the data available (e.g., external variables and length of historical data). The estimated model can then be directly used to predict in a reliable and accurate way the measure across the upcoming week. Different types of models (times-series, Multiple Linear Regression—MLR, etc.) are considered and the best-fitted one is selected and parameterized per case to achieve the best performance. The prediction models have been implemented as R scripts and RapidAnalytics processes.

The rules (implemented as a Symfony PHP web application) are expert knowledge-based algorithms aimed at giving suggestions for intelligent energy management. They consist of simple logic-based rules (most of them based on logical sentences) that can be implemented and used for better managing a building that is already equipped with a network of sensors. The rules are divided into four individual sections, depending on three fields of application:

- Building (management of occupancy, heating and cooling technical systems, indoor thermal comfort, air cooling through air-side economizer strategies);
- Building and RES production (management of the generation and on-site RES production and exploitation);
- Building, RES production and storage (management the operation of different energy flows towards energy cost reduction).

Each rule or a combination of them generates an Action Plan that is the suggestion for better managing the building with the purpose of decreasing its energy consumption.

## 4. IoT-Based System

A web-based system was implemented, integrating the above-mentioned architecture. An important function of the tool is the immediate and complete virtual distribution on the Internet of the energy consumption in buildings. Thus, the user can be constantly updated on the energy consumption and other indicators (energy cost, CO_2_ emissions, etc.) wherever located, always with the ease of use of the website.

On the first level, the proposed system collects, analyses and presents data amongst four major groups of indicators, facilitating the energy management (Table 3).

- The first group consists of five indicators and focuses on the building’s energy consumption, either electricity or fossil fuels, which is directly compared with the building’s surface. It includes both data from realized consumptions and projections for future ones (I_GBT-11_, I_GBT-12_, I_GBT-13_). The other two indicators that constitute the consumption group are a little bit more detailed (I_GBT-14_, I_GBT-15_). This group of indicators provides valuable information to the users groups, both for monitoring and taking action plans.
- The second group of indicators is more technical and focuses on the power efficiency, in order to address any malfunctions (I_GBT-21_, I_GBT-22_).
- The third group emphasizes on the energy management’s environmental impact, through calculating the damage that is done or is avoided, depending on the way that the consumed energy is produced. The group of indicators varies with the location of the building, as well as its features (I_GBT-31_, I_GBT-32_, I_GBT-33_).
- The fourth and last group of indicators, the monetary one, deals with the economic impact. It uses the consumptions’ data and projection and calculates the relevant cost (I_GBT-41_, I_GBT-42_, I_GBT-43_).

The values of indicators are based on virtual sensors, namely synthetic sensors whose date is obtained from existing sensors. For example, a virtual sensor can be created to obtain the total energy consumption of a building per square meter that can have several physical sensors for monitoring the data consumptions of the different sections.

At a second level, simple rules can be applied, giving suggestions for the improvement of energy management related to the management of the occupancy, heating and cooling technical systems, indoor thermal comfort, air cooling through air-side economizer strategies, generation and on-site RES production and exploitation, etc.

**Figure 3:**
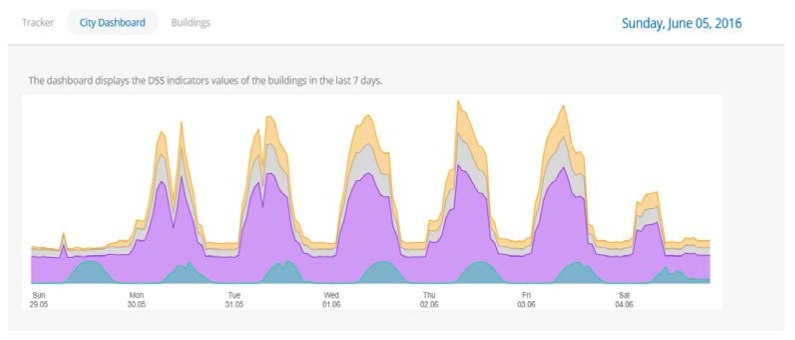
Energy assets.

Once the user has logged in, the main screen provides some general information about the buildings and their energy performance (Figure 3). For the energy consumption monitoring of a specific category of assets, we are allowed, by choosing particular filters, to export relevant diagrams for the energy consumption. We can choose which asset(s) we wants to review, and as to what energy or environmental indicator.

**Figure 4:**
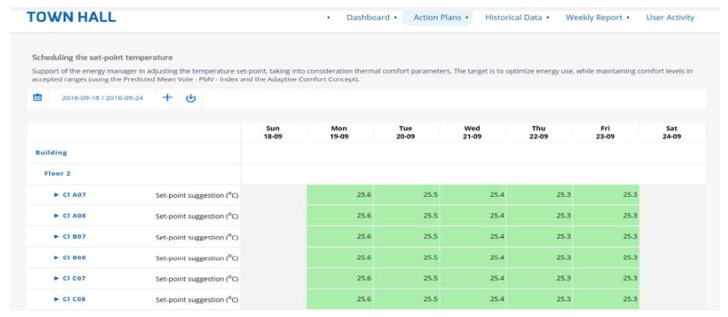
Front-end environments.

Moreover, the user can chose among different options, such as action plans, historical data, weekly report and user activity (Figure 4). Another useful feature of the system is the fact that it provides the user with information related to the parameters affecting the action plans and the buildings. That way, the user can conclude whether there is any problem with the sensors and validate the suggestions provided by the algorithms of the system.

The system can be appropriately customized to the users’ requirements and building characteristics. All aspects of the system are open sourced. The code for data capturing modules, semantic framework, prediction models and rules are showcased in a distinct repository in order to be easily reusable [46].

## 5. Pilot Appraisal

### 5.1. Impact Assessment Methodology

The general framework for the assessment of the impact is based on the comparison of the energy consumption before and after the real implementation of the action plans (pre-action vs. post-action). The general framework includes the following four phases [47]:

- Pre-action phase: The energy consumption can be assessed by means of inverse models or forward models. The inverse models are built through the real-time data collection related both to climate and users (input data) and to historical energy consumption (output data). The forward models are fed by data related to climate, users, equipment, lighting (input data) and by building features (fixed parameters). The historical energy consumption data are then used to calibrate the model.
- Pre action tailoring with post-action input data: In order to make the comparison between pre-action and post-action energy consumption consistent, the models (inverse or forward) developed in the previous phase need to be tailored. This means that the calibrated models are tailored considering the boundary conditions (climate and user) occurring in the post action phase.
- Post-action phase: In the post-action phase, the energy consumption can be assessed in two different ways: modification of the forward model through the application of the inference rules (when the implementation of the action plans is simulated); energy monitoring (when the action plans are actually implemented).
- Impact assessment: The impact of the action plans application can be assessed in four different ways: inverse model vs. inverse model; monitoring vs. forward model; monitoring vs. inverse model; forward model vs. forward model.

### 5.2. Building’s Characteristics

The presented IoT-based system was applied to the building premises of the Decision Support System Laboratory of the School of Electrical and Computer Engineering, National Technical University of Greece (DSS Lab). The pilot operation aimed at the combination and interconnection of an advanced IoT-based system, with smart automation systems and smart technologies and equipment (smart meters, sensors, etc.).

The DSS Lab building is located in Athens, Greece. It is an old building, which was built in 1979. The building is covering an area of 244 m^2^ and consists of two floors, where the offices of the employees are situated, and also one meeting room. Normal working hours for the offices are 10:00 a.m. to 8:00 p.m. The building is occupied by 40 employees on an average day.

**Figure 5:**
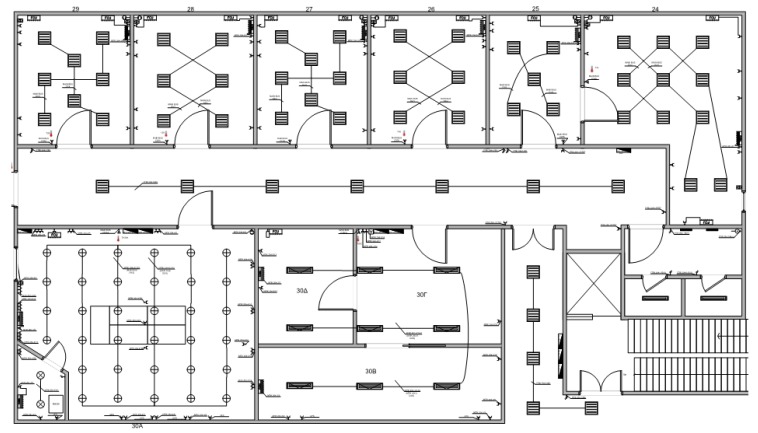
DSS Lab Plan, Floor 0.

In this context, equipment was placed across the premises of the DSS Lab and a pilot connection to the proposed IoT-based system was implemented (Figure 5). A number of sensors and energy meters were placed across the premises and the areas of the lab, in order to provide useful data to the system. More specifically, the installed equipment is used to provide measurements about indoor and outdoor temperature, humidity, lighting function and energy consumption for each area (47 data streams in total), as follows:

- 2 master generators,
- 7 relay modules,
- 9 I/O modules,
- 22 energy meter,
- 6 environmental sensors,
- 1 temperature sensor.

### 5.3. Baseline Scenario

The building is supplied by medium voltage electricity, which is converted to low voltage. In the case of black out, the building makes use of a back-up generator that operates on diesel. Electricity is used to cover the needs for heating, cooling, lighting, etc.

The total electricity consumptions for the baseline year 2014 was 87.2 KWh (January 9%; February 8.3%; March 7.6%; April 7.8%; May 7.9%; June 9.6%; July 11%; August 6.6%; September 8.4%; October 7.6%; November 7.4%; and December 8.8%). The figures are derived from the historical energy data and the monitored data. The total CO_2_ emissions are estimated at 86 tnCO_2_ per year and the energy cost 11,300 €.

### 5.4. Impact Analysis

The “monitoring vs. forward model” has been applied for the impact analysis. In this case, the tailored forward model developed in the phase “pre action tailoring with post-action input data” is capable of estimating the energy consumption according to different boundary conditions without considering the effect of the action plans. The resulting simulated energy consumption can be considered as a baseline. In order to assess the actual effect of the action plans (impact), a comparison between the actual energy consumption consequent to the application of the action plans and the output of the forward baseline model is carried out.

During the pilot implementation, the following action plans were applied. The building’s data (indoor air temperature and energy consumption), weather conditions (outdoor air temperature), energy prices and end-users’ behaviour data concerning thermal comfort are integrated. The rules are automatically generated during the configuration of the system:

- Optimising the boost time of the heating/cooling system taking into account the forecasting of the indoor air temperature and the occupancy levels of the building.
- Scheduling the set-point temperature by taking into consideration thermal comfort of the occupants. The users were able to choose different schedules and set point temperatures for each office.

**Figure 6:**
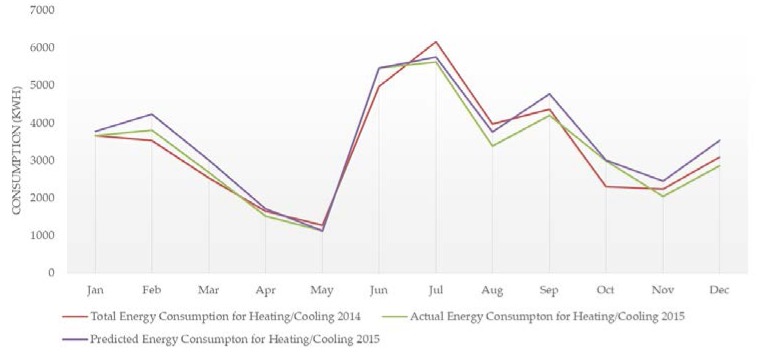
Energy consumption data (2014 and 2015)—1st year of operation.

**Figure 7:**
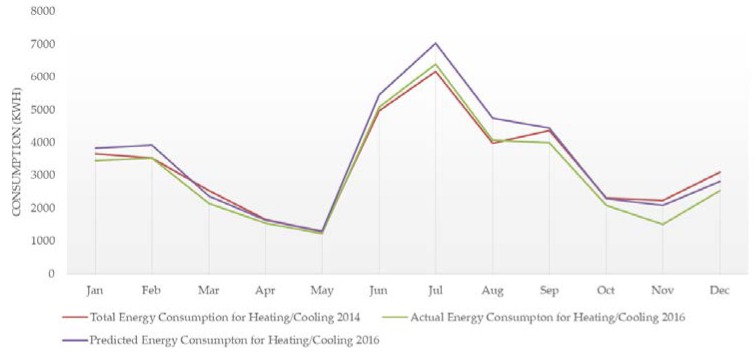
Energy consumption data (2014 and 2016)—2nd year of operation.

The total energy consumption for heating/cooling in 2014 (baseline), as well as the actual/predicted energy consumption for heating/cooling during the period 2015–2016 (pilot operation period), are presented in Figure 6 and Figure 7. The predicted energy consumption is based on the energy consumption for heating/cooling in 2014 and the degree days. The reduction of energy consumption for heating/cooling is estimated at 8.1% in 2015 and 10.9% in 2016. As a result, a significant decrease of the building’s operating cost was achieved, estimated at 11.3%.

The results revealed the significant potential for energy savings through the installation and operation of the proposed IoT-based system. Taking into consideration the initial cost for the installation and operation of the system, the average annual energy savings as derived from the system pilot application and the annual maintenance cost, the payback period is estimated at two years approximately.

### 5.5. Future Prospects

The idea of the Smart Buildings could be generalized, including additional application areas, such as the following:

- “Pillars” applications, focusing on the street and road lighting control by analysing the lamps’ failures and reports’ crucial data for the local authorities.
- “Electrical Vehicle” applications, processing data from electric vehicles charging stations, namely those parking spaces where electric vehicles supply equipment, is used to charge vehicles.

The above could provide local authorities with tools to manage their energy consumption in different categories of infrastructures encountered in Smart Cities.

Moreover, this extra information could be exploited in order to create more rules and visualizations that could further enhance the cognitive comprehension of each person interested in how the system operates and make it user friendly for further sustainability. The Smart Appliances REFerence (SAREF) ontology could also be used as an input for the ontology generation [48].

The deployment of innovative award incentive mechanisms for the energy end-users could also enable behavioural energy efficiency. The innovative aspect of such mechanisms is that the energy end-users will be able to automatically generate their own coins (through a virtual energy currency), by reducing their energy consumption.

## 6. Conclusions

ICT-based solutions that exploit Internet of Things (IoT) technologies can contribute significantly to energy saving, by motivating and supporting behavioural change of the buildings’ occupants. In this context, the proposed IoT-based system facilitates energy end-users to know how much energy is consumed in total and what is the contribution of the specific end-user and other peers to that, as well as get personalized recommendations of actions for energy conservation and load shifting, along with an estimation of their impact on energy use and user comfort.

The main aim was to provide a flexible, easy to expand and easily customizable system from an administrator (all permissions) and from a user perspective (view customization) scalable, ICT platform. The system uses data sensors that are installed in the building and measure real-time data as regards consumption of systems and appliances, occupancy data, behavioural data, set points, system setting, etc. It simplifies the complexity of the information gathered by those systems, and put it in the hands of energy end-users (buildings’ occupants), in context (e.g., end-users know how to improve the building behaviour when he/she is in the building, performing a specific action). Moreover, it could be used from the city authorities for the monitoring and management of the city’s energy status in buildings.

Semantic Web technologies could actually play a key role in the rapid development of various aspects of the Smart City infrastructure where various other research areas could come into play. The intersection of Web Services, Semantic Web and energy management could help city authorities towards a smoother transition to the future of cities.

## Figures and Tables

**Table 1:** Operating process.

	**DATA INTEGRATION**				
**Pillars**	❶	❷	❸	❹	❺
	Building‘s data	Energy Production	Energy prices	Weather data	End-Users’ Behaviour
**Input**	Electricity consumption Indoor temperatures Presence detectors	Renewable Energy (PV)	Real time market data	Temperature Weather conditions	Comfort feeling Schedule/location
**Decision supported**	**PREDICTION MODELS/RULES**				
	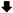				
	**ACTION PLANS SUGGESTION**				
**Pillars**	ENVIRONMENT-“WE”		USER-“I”		
**Benefits**	CO_2_ emissions reduction Energy consumption cut down		Experience improvement Energy Cost		

**Table 2:** Existing systems and pillars applied.

Existing Systems	Reference	Pillars Applied
Fotopoulos et al. (2017)	[21]	❶ + ❷ + ❹ + ❺
Terroso-Saenz et al. (2017)	[22]	❶ + ❷ + ❹ + ❺
Schneider Electric StruxureWare™	[23]	❶ + ❺
Honeywell Attune Advisory Services	[24]	❶ + ❷
Siemens Synco™	[25]	❶ + ❷
Cylon Energy solution	[26]	❶ + ❷
eSight	[27]	❶ + ❷ + ❺
Enerit Systematic Energy Management Software	[28]	❶ + ❷
DEXCell Energy Manager	[29]	❶ + ❸ + ❹
Predictive Energy Optimization™	[30]	❶ + ❸ + ❹
Ameresco‘s Intelligent Solutions (AIS)	[31]	❶ + ❷
Loop Energy Saver, Origami Energy, NUUKA, OPTIWATTI, Plugwise, SMARKIA, Bidgely, Enetics and PlotWatt	[32,33,34,35,36,37,38,39,40]	❶ + ❷ + ❹ + ❺

**Table 3:** Indicators.

Index	Indicator	
	Title	Unit
I_GBT-11_	Electricity per floor area	KWh/m^2^
I_GBT-12_	Electricity per use per area	kWh/m^2^ for lighting, cooling, other uses
I_GBT-13_	Fuel used for heating per floor area	lt/m^2^ (either Heating oil or Natural Gas)
I_GBT-14_	Electrical Energy per floor area and user	kWh/m^2^/user or kWh/m^2^/manhour
I_GBT-15_	Fuel used for heating per floor area and user	lt/m^2^/user
I_GBT-21_	Electrical Power	kW (constant metering)
I_GBT-22_	Electrical Power Factor	cosφ
I_GBT-31_	CO_2_ emissions for Electricity per floor area	tn/m^2^
I_GBT-32_	CO_2_ emissions for Heating per floor area	Lt/m^2^
I_GBT-33_	Produced electricity by RES (PVs)	kWh
I_GBT-41_	Cost of Electricity per floor area,	€/m^2^
I_GBT-42_	Cost of Fuel used for Heating per floor area	€/m^2^
I_GBT-43_	Monthly calculation of the electricity cost and potential projection through correlation with degree days and users	-
